# Assessing the predictive value of the controlling nutritional status score on all-cause mortality during hospitalization in patients with acute decompensated heart failure: a retrospective cohort study from Jiangxi, China

**DOI:** 10.3389/fnut.2024.1392268

**Published:** 2024-07-05

**Authors:** Xin Huang, Jiajun Qiu, Maobin Kuang, Chao Wang, Shiming He, Changhui Yu, Guobo Xie, Guotai Sheng, Yang Zou

**Affiliations:** ^1^Jiangxi Medical College, Nanchang University, Nanchang, Jiangxi, China; ^2^Jiangxi Cardiovascular Research Institute, Jiangxi Provincial People’s Hospital, The First Affiliated Hospital of Nanchang Medical College, Nanchang, Jiangxi, China; ^3^Jiangxi Provincial Geriatric Hospital, Jiangxi Provincial People’s Hospital, The First Affiliated Hospital of Nanchang Medical College, Nanchang, Jiangxi, China; ^4^Department of Cardiology, Jiangxi Provincial People’s Hospital, The First Affiliated Hospital of Nanchang Medical College, Nanchang, Jiangxi, China

**Keywords:** predictive value, controlling nutritional status score, acute decompensated heart failure, CONUT score, ADHF, in-hospital mortality

## Abstract

**Objective:**

Nutritional status is closely associated with the prognosis of heart failure. This study aims to assess the relationship between the Controlling Nutritional Status (CONUT) score and in-hospital mortality among patients with acute decompensated heart failure (ADHF) in Jiangxi, China.

**Methods:**

A retrospective cohort study was conducted. Multivariable Cox regression models and restricted cubic spline regression were employed to evaluate the relationship between the CONUT score and in-hospital mortality in ADHF patients from Jiangxi, China. The predictive value of the CONUT score for in-hospital mortality in ADHF patients was analyzed using receiver operating characteristic curves. Subgroup analyses were performed to identify risk dependencies of the CONUT score in specific populations.

**Results:**

The study included 1,230 ADHF patients, among whom 44 (3.58%) mortality events were recorded. After adjusting for confounding factors, a positive correlation was found between the CONUT score and the risk of in-hospital mortality in ADHF patients. Restricted cubic spline regression analysis indicated a non-linear relationship between the CONUT score and the risk of in-hospital mortality in ADHF patients, estimating a rapid increase in mortality risk when the CONUT score exceeded 5. Receiver operating characteristic analysis demonstrated a good predictive value of the CONUT score for all-cause mortality events in ADHF patients [area under the curve = 0.7625, optimal threshold = 5.5]. Additionally, a relatively higher risk associated with the CONUT score was observed in male patients and those with concomitant cerebral infarction.

**Conclusion:**

This study reveals a positive correlation between the CONUT score and the risk of in-hospital mortality in ADHF patients. Based on the findings of this study, we recommend maintaining a CONUT score below 5 for patients with ADHF in Jiangxi, China, as it may significantly contribute to reducing the risk of in-hospital all-cause mortality.

## Introduction

Heart failure (HF) is an increasingly grave global public health challenge, with an estimated prevalence exceeding 37.7 million individuals (1). Despite advancements in the management and treatment of HF due to rapid developments in medical technology in recent years, the annual mortality rate remains high (2–5). Most hospital admissions for HF patients are associated with acute decompensated heart failure (ADHF) (6, 7), which refers to a sudden exacerbation of HF symptoms and signs caused by various factors (8, 9), manifesting as hemodynamic abnormalities and end-organ damage (10, 11). The in-hospital mortality rate for ADHF varies between 4% and 15.8%, imposing significant physical and emotional burdens on patients and their families (12–15). Early identification of risk factors for in-hospital mortality in ADHF patients is crucial for improving their short-term prognosis.

Malnutrition is notably common among ADHF patients, likely related to fluid retention causing intestinal edema, decreased secretion of growth peptides, leading to anorexia, malabsorption, and inflammatory responses (16, 17). Evidence suggests a link between nutritional status and the prognosis of ADHF (18–21). Consequently, incorporating nutritional status into the overall assessment of patients to mitigate malnutrition-related risks is essential. Various nutritional assessment scales have been developed by clinicians and researchers for more accurate assessment, such as the Mini Nutritional Assessment, handgrip strength, Subjective Global Assessment, and the Controlling Nutritional Status (CONUT) score (22–25). Unlike other nutritional scores, the CONUT score relies mainly on objective values of serum albumin (ALB), total lymphocyte count (TL), and total cholesterol (TC), and is primarily used to evaluate the prognosis of cancer, coronary artery disease, and patients undergoing dialysis (26–30). A recent retrospective cohort study from Japan demonstrated that the CONUT score could assess the in-hospital all-cause mortality risk in Japanese ADHF patients (31). However, the predictive value of the CONUT score for in-hospital mortality in ADHF patients remains unclear, particularly in the Chinese population. Given the high mortality rate associated with ADHF, early clarification of the predictive value of the CONUT score for all-cause mortality during hospitalization in ADHF patients could be highly beneficial. To address this, the current study aims to assess the predictive value of the CONUT score for in-hospital all-cause mortality among ADHF patients in a cohort from Jiangxi, China.

## Methods

### Study design and data source

**FIGURE 1:**
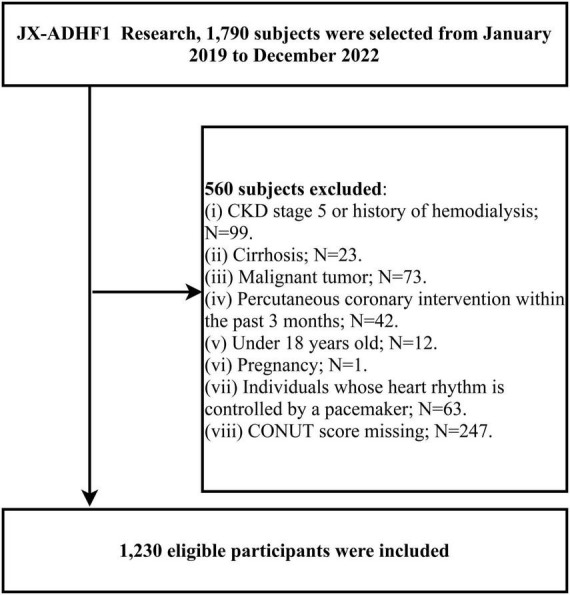
Flow chart for inclusion and exclusion of study participants.

This research utilized data from the JX-ADHF1 (Jiangxi-Acute Decompensated Heart Failure 1) study, a retrospective observational study that consecutively included 1,790 ADHF patients admitted to the Jiangxi Provincial People’s Hospital between January 2019 and December 2022. The definition of ADHF was based on the latest European Society of Cardiology (ESC) guidelines for acute and chronic heart failure available at the time. In this study, we excluded participants with the following characteristics: (1) those with stage 5 chronic kidney disease or a history of hemodialysis (n = 99), and those with cirrhosis (n = 23); (2) those with malignancies (n = 73); (3) those who underwent percutaneous coronary intervention within the past three months (n = 42); (4) those under 18 years of age (n = 12); (5) pregnant participants (n = 1); (6) those with pacemakers (n = 63); and (7) those with missing CONUT score information (n = 247): missing TL (n = 25), ALB (n = 25), TC (n = 197). Ultimately, the study included 1,230 participants. The detailed screening process for the study population was shown in Figure 1.

### Ethical approval

The JX-ADHF1 study adhered to the ethical principles of the Declaration of Helsinki. The use of study data was approved with informed consent from the participants, and the research protocol was authorized by the Ethics Review Committee of Jiangxi Provincial People’s Hospital (IRB: 2024-01).

### Baseline information measurement and assessment

Participants were received by medical staff upon admission and their gender, age, body mass index (BMI), smoking and drinking status, New York Heart Association (NYHA) functional classification at admission, comorbidities [hypertension, diabetes, cerebral infarction, coronary heart disease, pulmonary infection, atrial fibrillation (PASP)], as well as blood pressure, and most recent echocardiogram results were recorded in the medical record system (including left ventricular ejection fraction (LVEF), mitral regurgitation, tricuspid regurgitation and PASP data). In addition, we recorded information on the medications administered to the subjects during their hospitalization, including furosemide, spirolactone, angiotensin-converting enzyme inhibitors (ACEI)/angiotensin receptor inhibitors (ARB)/angiotensin receptor neprilysin inhibitors (ARNI), beta-blockers, digitalis, sodium-dependent glucose transporters 2 (SGTL-2), antiplatelet agent and lipid-lowering treatment information.

Laboratory parameters were measured within 24 hours after admission in a standard laboratory using an automatic analyzer, including N-Terminal Pro-Brain Natriuretic Peptide (NT-proBNP), white blood cell count (WBC), hemoglobin (Hb), neutrophil count (NEUT), TL, ALB, alanine aminotransferase (ALT), aspartate aminotransferase (AST), creatinine (Cr), TC, triglycerides (TG), low-density lipoprotein cholesterol (LDL-C), and high-density lipoprotein cholesterol (HDL-C). It should be noted that liver enzyme-related parameters and lipid parameters were determined by venous blood drawn on an empty stomach upon admission or the next morning.

The CONUT score was defined based on ALB, TC, and TL, categorized as no malnutrition (0–1), mild malnutrition (2–4), moderate malnutrition (5–8), and severe malnutrition (9–12); detailed information was provided in Table 1.

**TABLE 1:** Definition of Controlling Nutritional Status (CONUT) score.

Parameter	Malnutritional state			
	None	Light	Moderate	Severe
ALB (g/L)	≥ 35.0	30.0–34.9	25.0–29.9	< 25.0
Score	0	2	4	6
TL (× 10^9^/L)	≥ 1.60	1.20–1.59	0.80–1.19	< 0.80
Score	0	1	2	3
TC (mg/dl)	≥ 180	140–179	100–139	< 100
Score	0	1	2	3
Total CONUT score	0–1	2–4	5–8	9–12

ALB, serum albumin; TL, total lymphocyte count; TC, total cholesterol level, Total CONUT score = ALB score + TL score + TC score.

### Outcome definition

The start time of follow-up was defined as the admission time of patients with ADHF, and the study primary end point was death from any cause during hospitalization, with the secondary endpoint event being death from cardiovascular causes.

### Statistical analysis

The baseline information of the study population was described using continuous variables as means [standard deviations] or medians (interquartile range: 25th–75th percentiles) and categorical variables as frequencies (%). Variance analysis or the Kruskal-Wallis H test was used for intergroup comparisons of continuous data, while the chi-square test was employed for intergroup comparisons of categorical data. Kaplan-Meier analysis was utilized to plot cumulative survival curves for different CONUT score groups.

To estimate the risk of high CONUT scores relative to low scores for the outcome indicator, multivariable Cox regression models were used to examine the relationship between CONUT scores and all-cause/cardiovascular mortality during hospitalization in ADHF patients, reporting adjusted hazard ratios (HRs) and 95% confidence intervals (CIs). Model 1 adjusted for gender, age, hypertension, diabetes, cerebral infarction, and coronary heart disease; Model 2 further adjusted for NYHA classification, LVEF, systolic blood pressure (SBP), diastolic blood pressure (DBP), drinking, and smoking status on the basis of Model 1; Model 3 further included WBC, AST, TG, HDL-C, LDL-C, NT-proBNP, and Cr adjustments based on Model 2. Model 4 further adjusted for etiology of ADHF, AF, Pulmonary infection, Hb, BMI and anti-heart failure treatment. Restricted cubic spline regression was employed to assess the dose-response relationship between CONUT scores and all-cause/cardiovascular mortality during hospitalization in ADHF patients.

Receiver operating characteristic (ROC) curves were used to evaluate the predictive accuracy of the CONUT score and its components for all-cause mortality during hospitalization in ADHF patients, identifying the best threshold, sensitivity, and specificity. The DeLong test was used to compare whether there was a difference in the area under the curve (AUC) values between the CONUT score and its components. Finally, stratified analysis was conducted based on age, gender, NYHA classification, LVEF, and the presence of hypertension, diabetes, cerebral infarction, and coronary heart disease. Likelihood ratio tests were used to compare differences between subgroups and to assess the presence of interaction effects.

For all tests, statistical significance was set at *P* < 0.05. All analyses were conducted using R language version 3.4.3 and Empower(R) version 2.0.

## Results

### Baseline characteristics of participants

Among the 1,230 eligible adult participants, there were 724 males and 506 females, with an average age of 68 years. Based on the subjects’ disease onset characteristics and chronic heart failure history, we summarized the causes of these patients into the following nine categories: ischemic cardiomyopathy, hypertension, non-ischemic cardiomyopathy (including dilated cardiomyopathy, restrictive cardiomyopathy, hypertrophic cardiomyopathy, stress cardiomyopathy, systemic lupus erythematosus cardiomyopathy, diabetic cardiomyopathy and alcoholic cardiomyopathy), valvular disease, arrhythmia, acute myocarditis, congenital heart disease, pulmonary heart disease and other reasons (includes pericardial disease, hypothyroidism, anemic heart disease, severe infections, rapid progression of other systemic diseases, and unknown etiologies). As can be seen in Table 2, the main etiologies of ADHF patients in the current cohort were ischemic cardiomyopathy, non-ischemic cardiomyopathy and valvular disease.

**TABLE 2:** The most common etiologies of acute decompensated heart failure.

Etiology of ADHF	All (N = 1230)
Ischemic cardiomyopathy	380 (30.89%)
Hypertension	120 (9.76%)
Non-ischemic cardiomyopathy	305 (24.80%)
Valvular disease	234 (19.02%)
Arrhythmia	74 (6.02%)
Acute myocarditis	8 (0.65%)
Congenital heart disease	28 (2.28%)
Pulmonary heart disease	63 (5.12%)
Other reasons	18 (1.46%)

Table 3 presents the baseline characteristics of the participants according to the four categories of CONUT scores. The results indicated significantly statistical differences in gender, age, BMI, LVEF category, NYHA classification, tricuspid regurgitation, PASP, DBP, WBC, NEUT, TG, TL, ALB, TC, HDL-C, LDL-C, AST, Cr, NT-proBNP, HF treatment with furosemide, spironolactone, ACEI/ARB/ARNI, digitalis and beta-blockers, complicated with pulmonary inflammation, and all-cause mortality rate across different CONUT score groups. Participants with higher CONUT scores generally had lower levels of TL, ALB, TC, Hb, HDL-C, LDL-C, but higher levels of PASP, AST, Cr, NT-proBNP (all *P* < 0.05). In addition, patients with high CONUT scores had a higher probability of having combined pulmonary inflammation, HF with preserved ejection fraction and tricuspid regurgitation, and a lower probability of having used furosemide, spironolactone, ACEI/ARB/ARNI, and beta-blockers.

**TABLE 3:** The malnutrition status defined by CONUT score shows the baseline characteristics of the subjects.

	Malnutritional state (CONUT score)				P-value
	None (0–1)	Light (2–4)	Moderate (5–8)	Severe (9–12)	
No. of subjects	176	569	424	61	
**Gender**					0.014
Female	91 (51.70%)	231 (40.60%)	163 (38.44%)	21 (34.43%)	
Male	85 (48.30%)	338 (59.40%)	261 (61.56%)	40 (65.57%)	
Age (years)	64.00 (53.00–72.00)	69.00 (59.00–77.00)	74.00 (64.75–81.00)	73.00 (65.00–83.00)	< 0.001
**Comorbidities**					
Hypertension (n,%)	75 (42.61%)	244 (42.88%)	184 (43.40%)	20 (32.79%)	0.470
Diabetes (n,%)	47 (26.70%)	148 (26.01%)	114 (26.89%)	17 (27.87%)	0.983
Cerebral infarction (n,%)	22 (12.50%)	98 (17.22%)	70 (16.51%)	14 (22.95%)	0.253
CHD (n,%)	57 (32.57%)	172 (30.34%)	147 (34.67%)	16 (26.23%)	0.381
AF	60 (34.09%)	235 (41.30%)	183 (43.16%)	23 (37.70%)	< 0.207
Pulmonary infection	30 (17.05%)	158 (25.77%)	194 (45.75%)	38 (62.30%)	< 0.001
**NYHA classification (n,%)**					< 0.001
III	141 (80.11%)	425 (74.69%)	270 (63.68%)	31 (50.82%)	
IV	35 (19.89%)	144 (25.31%)	154 (36.32%)	30 (49.18%)	
SBP (mmHg)	127.81 (23.06)	129.03 (23.76)	128.45 (26.20)	120.56 (26.23)	0.087
DBP (mmHg)	76.39 (15.85)	77.22 (15.21)	75.16 (16.28)	69.92 (14.06)	0.003
BMI	24.39 (3.71)	23.35 (4.07)	22.39 (3.77)	21.31 (4.77)	0.003
**LVEF category**					0.038
HFrEF	53 (30.64%)	171 (31.55%)	102 (25.63%)	11 (19.64%)	
HFmrEF	36 (20.81%)	134 (24.72%)	121 (30.40%)	12 (21.43%)	
HFpEF	84 (48.55%)	237 (43.73%)	175 (43.97%)	33 (58.93%)	
Mitral regurgitation	151 (85.80%)	473 (86.63%)	358 (86.89%)	50 (87.72%)	0.979
Tricuspid regurgitation	139 (78.98%)	495 (90.66%)	381 (92.48%)	52 (91.23%)	< 0.001
Pulmonary artery systolic pressure	44.00 (32.00–55.00)	45.00 (37.00–55.00)	49.00 (40.00–58.00)	49.00 (46.50–60.50)	0.003
WBC, (× 10^9^/L)	6.50 (5.50–7.76)	5.80 (4.80–7.48)	6.10 (4.60–8.29)	7.80 (5.10–12.41)	< 0.001
Hb	133.45 (19.17)	128.14 (19.97)	115.12 ± 23.69	105.95 ± 22.97	<0.001
NEUT, (× 10^9^/L)	4.01 (3.10–4.83)	3.93 (3.00–5.30)	4.50 (3.30–6.40)	6.40 (4.10–11.29)	< 0.001
TL, (× 10^9^/L)	1.75 (1.60–2.10)	1.20 (0.90–1.50)	0.80 (0.59–1.10)	0.60 (0.40–0.80)	< 0.001
Alb (g/L)	39.21 (2.67)	37.40 (3.78)	32.47 (3.67)	25.27 (3.18)	< 0.001
ALT (U/L)	23.00 (16.00–41.00)	21.00 (14.00–37.00)	20.00 (13.00–35.00)	21.00 (12.00–80.00)	0.178
AST (U/L)	24.00 (19.00–34.00)	25.00 (19.00–37.00)	26.00 (19.00–40.00)	30.00 (20.00–125.00)	0.027
Cr (umol/L)	78.00 (64.00–101.00)	85.00 (66.00–113.00)	96.00 (73.00–141.00)	122.00 (77.00–193.00)	< 0.001
TG(mmol/L)	1.56 (1.18–2.07)	1.18 (0.91–1.54)	1.04 (0.82–1.30)	1.08 (0.73–1.61)	< 0.001
TC(mmol/L)	4.85 (0.87)	3.94 (0.95)	3.34 (0.88)	2.84 (0.68)	< 0.001
HDL-C (mmol/L)	1.11 (0.26)	1.06 (0.30)	0.93 (0.26)	0.74 (0.29)	< 0.001
LDL-C (mmol/L)	3.16 (0.78)	2.46 (0.82)	2.05 (0.75)	1.66 (0.51)	< 0.001
NT-proBNP (pmol/L)	2739.50 (1548.75–4019.50)	3257.00 (1950.00–5354.00)	4466.50 (2887.25–6278.50)	4617.00 (2568.00–6851.00)	< 0.001
**Drinking status (n,%)**					0.094
No	154 (87.50%)	519 (91.21%)	402 (94.81%)	58 (95.08%)	
Occasional	10 (5.68%)	25 (4.39%)	13 (3.07%)	2 (3.28%)	
Regular	12 (6.82%)	25 (4.39%)	8 (1.89%)	1 (1.64%)	
Quit	0 (0.00%)	0 (0.00%)	1 (0.24%)	0 (0.00%)	
**Smoking status (n,%)**					0.366
No	146 (82.95%)	497 (87.35%)	370 (87.47%)	54 (88.52%)	
Occasional	7 (3.98%)	26 (4.57%)	17 (4.02%)	1 (1.64%)	
Regular	23 (13.07%)	41 (7.21%)	32 (7.57%)	6 (9.84%)	
Quit	0 (0.00%)	5 (0.88%)	4 (0.95%)	0 (0.00%)	
**Anti-heart failure treatment**					
Furosemide	160 (90.91%)	543 (95.43%)	401 (94.58%)	54 (88.52%)	0.034
Spirolactone	160 (90.91%)	521 (91.56%)	376 (88.68%)	45 (73.77%)	< 0.001
ACEI/ARB/ARNI	119 (67.61%)	363 (63.80%)	229 (54.01%)	18 (29.51%)	< 0.001
Beta-blockers	137 (77.84%)	462 (81.20%)	309 (72.88%)	38 (62.30%)	< 0.001
Digitalis	60 (34.09%)	250 (43.94%)	196 (46.23%)	29 (47.54%)	0.045
SGLT-2	24 (13.64%)	71 (12.48%)	47 (11.08%)	6 (9.84%)	0.760
Antiplatelet agent	89 (50.57%)	275 (48.33%)	206 (48.58%)	22 (36.07%)	0.259
Lipid-lowering therapy	107 (60.80%)	325 (57.12%)	238 (56.13%)	27 (44.26%)	0.162
All-cause death	1 (0.57%)	10 (1.76%)	22 (5.19%)	11 (18.03%)	< 0.001

CONUT, Controlling Nutritional Status; CHD: coronary heart disease; NYHA: New York Heart Association; LVEF, left ventricular ejection fraction; SBP, systolic blood pressure; DBP, diastolic blood pressure; TG, triglyceride; TC, total cholesterol; HDL-C, high-density lipoprotein cholesterol; LDL-C, low-density lipid cholesterol; Cr, creatinine; ALT, alanine aminotransferase; AST, aspartate aminotransferase; NEUT, neutrophil count; Alb, albumin; NT-proBNP, N-Terminal Pro-Brain Natriuretic Peptide; SGLT-2, sodium-dependent glucose transporters 2; AF, atrial fibrillation; HFrEF, heart failure with reduced ejection fraction (LVEF < 40%); HFmrEF, heart failure with mid-range ejection fraction (LVEF 40–49%); HFpEF, heart failure with preserved ejection fraction (LVEF ≥ 50%); ACEI, angiotensin-converting enzyme inhibitors; ARB, angiotensin receptor inhibitors; ARNI, angiotensin receptor neprilysin inhibitors.

**FIGURE 2:**
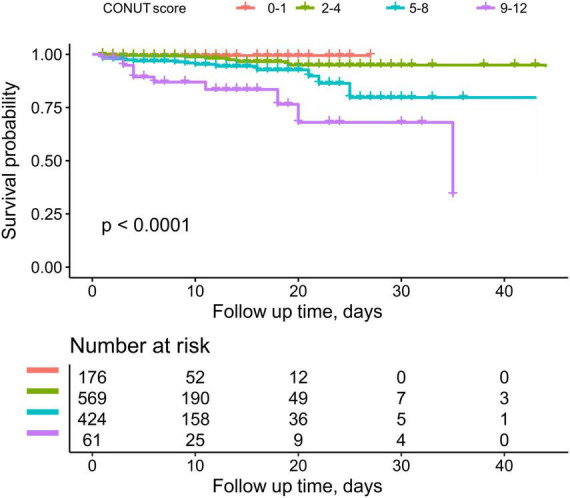
Cumulative survival rate curves of ADHF patients in different nutritional status groups.

During the observation period, 44 participants experienced death during hospitalization, of which 37 were classified as deaths from cardiovascular causes. Figure 2 depicts the trend of cumulative survival rates for ADHF patients, showing lower survival rates in the severe malnutrition group over time.

### Association between CONUT score and in-hospital mortality in ADHF patients in China

**FIGURE 3:**
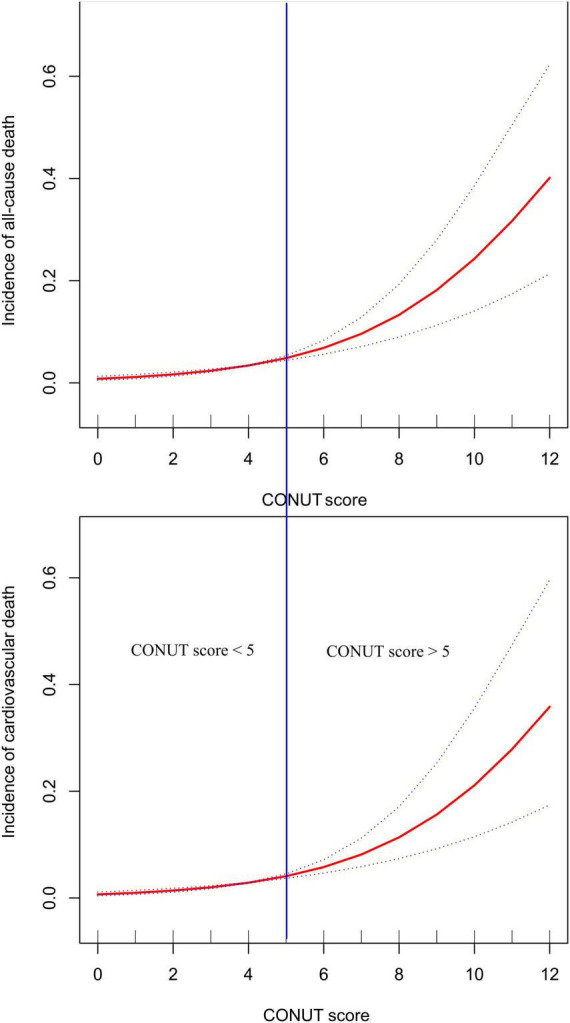
Fitting the dose-response relationship between CONUT score and all-cause mortality and cardiovascular mortality in ADHF patients with 3 knots restricted cubic spline.

Before establishing multivariable Cox regression models, all covariates underwent collinearity diagnostics. Variables such as ALT and TC with variance inflation factor values greater than 3 were considered to have high collinearity and were not included in subsequent adjustment models (Supplementary Table 1). Four progressively adjusted Cox regression models were established to assess the relationship between CONUT score and in-hospital mortality in ADHF patients (Table 4). It was found that in all models, the CONUT score were significantly and positively associated with both in-hospital all-cause mortality and cardiovascular death In the fully adjusted Model 4, each additional unit increase in the CONUT score was associated with a 25% (HR 1.25, 95% CI: 1.06,1.47) increase in the risk of all-cause mortality and a 22% (HR 1.22, 95% CI: 1.01,1.48) increased risk of cardiovascular death during hospitalization in ADHF patients. When treating the CONUT score as a categorical variable and using the no malnutrition category as the reference, the severe malnutrition category had the highest risk of in-hospital all-cause/cardiovascular mortality in ADHF patients. Additionally, it’s noteworthy that after fitting with smooth splines, we observed a potential threshold effect in the relationship between the CONUT score and all-cause/cardiovascular mortality during hospitalization in ADHF patients (Figure 3), estimating a rapid increase in mortality risk when the CONUT score exceeded 5.

**TABLE 4:** Multivariable Cox regression analysis of the relationship between CONUT score and in-hospital all-cause mortality in patients with acute decompensated heart failure.

	Hazard ratios (95% confidence interval)				
	Unadjusted model	Model 1	Model 2	Model 3	Model 4
**All-cause death**					
Conut score (continuous variable)	1.40 (1.25, 1.56)	1.40 (1.25, 1.57)	1.33 (1.16, 1.51)	1.24 (1.06, 1.44)	1.25 (1.06, 1.47)
**Conut score (categorical variable)**					
None (0–1)	Ref	Ref	Ref	Ref	Ref
Light (2–4)	2.41 (0.30, 19.04)	1.89 (0.23, 15.21)	1.78 (0.22, 14.67)	1.60 (0.16, 16.03)	1.43 (0.13, 15.26)
Moderate (5–8)	8.10 (1.09, 60.15)	6.06 (0.80, 45.64)	4.98 (0.65, 38.30)	3.23 (0.31, 33.42)	2.84 (0.25, 31.84)
Severe (9–12)	24.00 (3.08, 187.05)	18.59 (2.35, 147.25)	11.49 (1.37, 96.33)	5.79 (0.51, 66.13)	6.15 (0.49,76.58)
*P*-trend	< 0.0001	< 0.0001	< 0.0001	0.0201	0.0179
**Cardiovascular death**					
**Conut score (continuous variable)**					
	1.39 (1.24, 1.57)	1.39 (1.23, 1.58)	1.30 (1.12, 1.50)	1.20 (1.01, 1.42)	1.22 (1.01, 1.48)
**Conut score (categorical variable)**					
None (0–1)	Ref	Ref	Ref	Ref	Ref
Light (2–4)	2.10 (0.26, 16.86)	1.61 (0.20, 13.22)	1.49 (0.18, 12.56)	1.10 (0.12, 9.78)	1.08 (0.11, 10.13)
Moderate (5–8)	6.92 (0.92, 51.77)	5.17 (0.68, 39.36)	3.69 (0.47, 29.00)	1.79 (0.19, 16.85)	1.54 (0.15, 15.33)
Severe (9–12)	19.11 (2.40, 152.13)	14.60 (1.80, 118.46)	8.00 (0.91, 70.06)	3.10 (0.29, 32.92)	4.08 (0.34, 48.63)
*P*-trend	< 0.0001	<0.0001	0.0015	0.0137	0.0408

Model 1 adjusted for gender, age, hypertension, diabetes, cerebral infarction and CHD; Model 2 adjusted for model 1 + NYHA classification, LVEF, SBP, DBP, drinking status and smoking status; Model 3 adjust for: Model 2 + WBC, AST, TG, HDL-C, LDL-C, NT-proBNP and Cr. Model 4 adjust for: Model 3 + etiology of ADHF, AF, pulmonary infection, Hb, BMI and anti-heart failure treatment.

### Accuracy of CONUT score in identifying all-cause mortality events during hospitalization in ADHF patients

**FIGURE 4:**
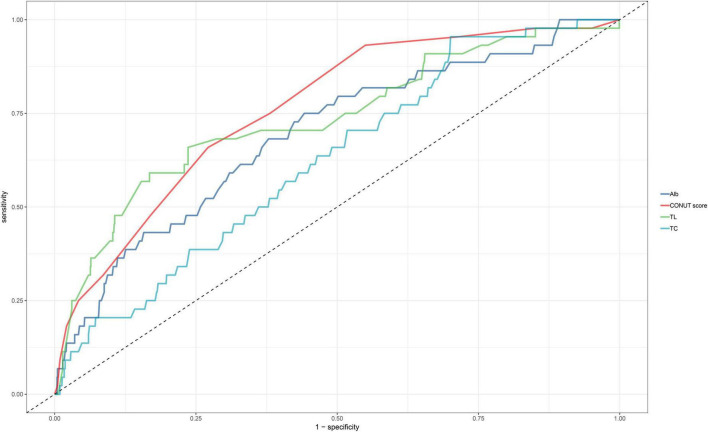
ROC analysis shows the predictive value of CONUT score and its components for all-cause mortality in patients with ADHF.

ROC curves were utilized to evaluate the ability of the CONUT score and its components (TL, ALB, and TC) to identify all-cause mortality during hospitalization in ADHF patients (Table 5). As depicted in Figure 4, the CONUT score demonstrated the highest AUC value for predicting all-cause mortality during hospitalization in ADHF patients compared to TL, ALB, and TC (AUC: CONUT score 0.7625, TL 0.7359, ALB: 0.6926, TC 0.6257; All DeLong *P* < 0.0001), with the optimal threshold identified as 5.5.

**TABLE 5:** Area under the receiver operating characteristic curve of the CONUT score and its components on in-hospital all-cause mortality in patients with acute decompensated heart failure.

	AUC	95%CI low	95%CI upp	Best threshold	Specificity	Sensitivity
CONUT score	0.7625	0.6947	0.8302	5.5000	0.7285	0.6591
TL*	0.7359	0.6514	0.8204	0.6644	0.8322	0.5909
Alb*	0.6926	0.6107	0.7746	35.0500	0.5582	0.7500
TC*	0.6257	0.5484	0.7029	4.2450	0.2993	0.9545

AUC, area under the curve; other abbreviations as in Table 1.

*P < 0.05, compare with CONUT score (Delong test).

### Subgroup analysis

Stratified analyses were conducted based on age, gender, NYHA classification, LVEF, and the presence of hypertension, diabetes, cerebral infarction, and coronary heart disease to explore potential specific populations affecting the relationship between the CONUT score and all-cause mortality during hospitalization in ADHF patients (Table 6). These parameters were stratified according to clinically common critical points. The results indicated that, except for the gender and history of cerebral infarction subgroups, there were no significant interactions between other subgroup factors and CONUT score-related in-hospital all-cause mortality. In the gender subgroup, compared to females, male ADHF patients had a significantly higher risk of CONUT score-related in-hospital all-cause mortality (HR: 1.34 vs 1.06). In the subgroup with a history of cerebral infarction, ADHF patients with concomitant cerebral infarction had a significantly higher risk of CONUT score-related in-hospital all-cause mortality compared to those without cerebral infarction (HR: 1.71 vs 1.05).

**TABLE 6:** Stratified analysis showed the relationship between CONUT score and in-hospital all-cause mortality in patients with acute decompensated heart failure in different age, gender, NYHA class, LVEF and whether combined with hypertension/diabetes/cerebral infarction/CHD.

Subgroup	Adjusted HR (95%CI)	*P* for interaction
Age (years)		0.8649
20–70	1.15 (0.87, 1.52)	
71–96	1.18 (0.95, 1.47)	
Gender		0.0335
Male	1.40 (1.15, 1.71)	
Female	1.03 (0.81, 1.31)	
NYHA		0.5769
III	1.50 (1.08, 2.09)	
IV	1.35 (1.10, 1.67)	
LVEF		0.6178
HFrEF (LVEF < 40%)	1.73 (1.09, 2.73)	
HFmrEF (LVEF 40–49%)	1.41 (0.66, 3.01)	
HFpEF (LVEF ≥ 50%)	2.09 (1.41, 3.08)	
Hypertension		0.9927
Yes	1.25 (1.02, 1.53)	
No	1.25 (0.98, 1.60)	
Diabetes		0.5678
Yes	1.46 (1.13, 1.90)	
No	1.30 (0.93, 1.82)	
Cerebral infarction		0.0317
Yes	1.66 (1.24, 2.22)	
No	1.16 (0.95, 1.41)	
CHD		0.2712
Yes	1.31 (1.09, 1.56)	
No	1.48 (1.28, 1.72)	

HFrEF, heart failure with reduced ejection fraction; HFmrEF, heart failure with mid-range ejection fraction; HFpEF, heart failure with preserved ejection fraction; other abbreviations as in Table 2. Models adjusted for the same covariates as in model 4 (Table 3), except for the stratification variable.

## Discussion

This study analyzed data from the JX-ADHF1 project spanning 2019–2022 to explore the association between nutritional status, as assessed by the CONUT score, and mortality during hospitalization in patients with ADHF. We found that the CONUT score could predict the risk of in-hospital all-cause/cardiovascular mortality in ADHF patients in the Jiangxi population of China. Notably, a CONUT score of 5 appeared to be a threshold effect point for poor in-hospital prognosis, with a rapid increase in mortality risk when the score exceeded this value.

ADHF is a worsening state of heart failure (12), primarily related to systemic congestion. Reduced cardiac output in ADHF can lead to insufficient perfusion of organs such as the gastrointestinal tract, lungs, liver, and kidneys, causing significant adverse effects (32, 33). Epidemiological studies have shown a high mortality rate during hospitalization for ADHF patients (34), highlighting the importance of assessing all-cause mortality risk at admission. Previous studies have indicated that a high CONUT score is an independent risk factor for all-cause mortality during hospitalization in ADHF patients in Japan and is closely associated with adverse outcomes in chronic heart failure patients in the United States (35). Considering national and ethnic differences and disease mechanisms, it was unclear whether this score would have the same effect in the Chinese ADHF population. Our study demonstrated a significant positive correlation between the CONUT score and all-cause mortality in ADHF patients in China, with every unit increase in the CONUT score increasing the risk of all-cause mortality during hospitalization by 24%, even after adjusting for confounding factors.

The CONUT score is composed of TL, ALB, and TC, with each component’s reduction and an increase in CONUT score associated with poorer nutritional status (36). Malnutrition and ADHF are interrelated in a bidirectional manner. Hormonal imbalances, metabolic disturbances, and immune dysregulation are key pathophysiological mechanisms in ADHF (33, 37–39). These changes lead to increased catecholamines, TNF-α, and natriuretic peptides, further accelerating the breakdown of ALB and fats, leading to malnutrition (40–44). Moreover, hypoalbuminemia exacerbates myocardial dysfunction by promoting pulmonary congestion, fluid retention, and myocardial edema (45–48). Hyperlipidemia is a risk factor for cardiovascular diseases, obesity, diabetes, and other metabolic disorders (49, 50), with various measures developed to reduce lipid levels (51–53). However, low lipid levels have been identified as a marker of increased mortality in heart failure patients (54–56), and reduced TC can increase the risk of death in ADHF patients (57). This lipid paradox might be related to inflammatory responses and nutritional status (58); decreased TC can elevate endotoxins, exacerbating inflammation (59, 60). Additionally, TL plays a protective role in cardiac homeostasis and response to injury (61–63). A reduction in TL count signifies a precarious state for the heart. The Seattle Heart Failure Model also identified TL as an independent predictor of heart failure prognosis (64), a finding further validated by our study.

Our analysis revealed a potential threshold effect in the dose-response relationship between the CONUT score and all-cause/cardiovascular mortality in ADHF patients. The risk of mortality increased rapidly when the CONUT score exceeded 5. Combined with the ROC analysis threshold result, we suggest that a CONUT score of 5 is significant for ADHF patients; scores exceeding this value indicate a potential for significant adverse events in the short term, warranting increased attention to improving nutritional status. Previous studies have also shown that nutritional status is related to the prognosis of cardiovascular diseases (36, 65–67), with patients suffering from moderate to severe malnutrition at a greater risk of adverse cardiovascular events, aligning with the findings of our study.

Our subgroup analysis observed some meaningful results: compared to female patients and those without cerebral infarction, male ADHF patients with cerebral infarction exhibited a relatively higher risk of in-hospital all-cause mortality associated with CONUT scores. This finding is consistent with the results summarized in the baseline characteristics table. Table 3 shows that as the severity of malnutrition increases, the proportion of male patients and those with cerebral infarction significantly rises. We have the following considerations for these particular results in male patients and those with cerebral infarction: (1) Compared to females, males generally tend to have poorer lifestyle habits, such as smoking, alcohol consumption, and worse dietary control (68), which can lead to various cardiometabolic diseases and exacerbate malnutrition (69–71). Additionally, patients with cerebral infarction often experience varying degrees of physical activity impairment, gastrointestinal dysfunction, and even feeding difficulties. These sequelae significantly aggravate the malnutrition in these patients (72). Therefore, the higher all-cause mortality related to CONUT scores in male and cerebral infarction patients may be associated with further malnutrition secondary to comorbidities. (2) From the perspective of CONUT score calculation, decreases in TL, ALB, and TC lead to higher CONUT scores, subsequently increasing the risk of adverse outcomes. In our study population, which mainly consisted of elderly individuals (mean age 68 years), postmenopausal women showed significantly reduced estrogen levels, leading to fat accumulation and elevated TC levels (73). It is noteworthy that high TC levels are associated with low CONUT scores, which might explain why the mortality risk related to CONUT scores is lower in females than in males. For patients with cerebral infarction, post-ischemic brain damage can easily trigger oxidative stress and inflammatory responses, disrupting the gut-brain axis and the hypothalamic-pituitary-adrenal axis, resulting in reduced TL production, decreased ALB synthesis, increased catabolism, and increased TC breakdown, leading to malnutrition (74–78).

### Study strengths and limitations

Strengths: (1) This study is the first to confirm the association between the CONUT score and in-hospital mortality in ADHF patients in the Chinese population, establishing its predictive value. (2) The CONUT score comprises easily accessible indicators, making it simple, convenient, and easily adoptable. (3) We identified population dependencies in the risk of in-hospital all-cause mortality related to the CONUT score in ADHF patients, highlighting the importance of identifying these specific populations for assessment.

Limitations: (1) The study population primarily comes from various regions in Jiangxi, and caution should be exercised when generalizing these findings to other regions in China and other countries. (2) As a retrospective study, it is subject to inherent statistical limitations, and our researchers had limited access to historical data, such as lipid-lowering medication use and family history. (3) This study assessed the impact of the CONUT score at admission on in-hospital mortality outcomes in ADHF patients, and the relationship between dynamic changes in the CONUT score and in-hospital adverse outcomes remains unclear, necessitating further research.

## Conclusion

The CONUT score is an independent predictor of all-cause mortality during hospitalization in patients with ADHF. Our study indicates that a CONUT score of 5 may represent a threshold effect point for the occurrence of all-cause mortality during hospitalization in ADHF patients. When the CONUT score exceeds 5, there is a rapid increase in the risk coefficient for all-cause mortality. Additionally, the relationship between the CONUT score and in-hospital mortality in ADHF patients shows a dependency on specific population subgroups, with male patients and those with a history of cerebral infarction requiring particular attention to their CONUT score results.

## Data availability statement

The raw data supporting the conclusions of this article will be made available by the authors, without undue reservation.

## Ethics statement

The studies involving humans were approved by the Ethics Review Committee of Jiangxi Provincial People’s Hospital. The studies were conducted in accordance with the local legislation and institutional requirements. The participants provided their written informed consent to participate in this study.

## Author contributions

XH: Formal analysis, Investigation, Software, Validation, Writing−original draft. JQ: Investigation, Software, Writing−original draft. MK: Formal analysis, Investigation, Software, Validation, Writing−original draft. CW: Investigation, Writing−review and editing. SH: Investigation, Writing−review and editing. CY: Investigation, Writing−review and editing. GX: Investigation, Writing−review and editing. GS: Conceptualization, Methodology, Project administration, Supervision, Writing−review and editing. YZ: Conceptualization, Investigation, Methodology, Project administration, Software, Supervision, Writing−review and editing.
