# Supplementary material for: Assessing the predictive value of the controlling nutritional status score on all-cause mortality during hospitalization in patients with acute decompensated heart failure: a retrospective cohort study from Jiangxi, China
Source: Front Nutr. 2024 Jul 5;11:1392268. doi: 10.3389/fnut.2024.1392268 (PMC11258027; doi:10.3389/fnut.2024.1392268)
Supplement: Supplementary file 1 [file Table_1.DOCX]

Supplementary Table 1: Collinearity diagnostics steps.

|  | VIF | | |
| --- | --- | --- | --- |
|  | Step 1 | Step 2 | Step 3 |
| CONUT score | 3.1 | 3.1 | 3.1 |
| Gender | 1.4 | 1.4 | 1.4 |
| Age | 1.6 | 1.6 | 1.6 |
| CHD | 2.2 | 2.2 | 2.2 |
| Hypertension | 1.5 | 1.5 | 1.5 |
| Diabetes | 1.3 | 1.3 | 1.3 |
| Cerebral infarction | 1.2 | 1.2 | 1.2 |
| AF | 1.3 | 1.3 | 1.3 |
| Pulmonary infection | 1.2 | 1.2 | 1.2 |
| NYHA classification | 1.2 | 1.2 | 1.2 |
| SBP | 1.7 | 1.7 | 1.7 |
| DBP | 1.5 | 1.5 | 1.5 |
| BMI | 1.3 | 1.3 | 1.3 |
| Drinking status | 1.9 | 1.9 | 1.9 |
| Smoking status | 1.9 | 1.9 | 1.9 |
| LVEF | 1.5 | 1.5 | 1.5 |
| WBC | 1.4 | 1.4 | 1.4 |
| Hb | 1.6 | 1.6 | 1.6 |
| ALB | 2.3 | 2.3 | 2.3 |
| ALT | 4.3 | 4.3 | NA |
| AST | 4.1 | 4.1 | 1.2 |
| Cr | 1.5 | 1.5 | 1.5 |
| TG | 2.3 | 1.2 | 1.2 |
| TC | 11.7 | NA | NA |
| HDL-C | 2.5 | 1.4 | 1.4 |
| LDL-C | 8.1 | 1.6 | 1.6 |
| NT-proBNP | 1.2 | 1.2 | 1.2 |
| Etiology of ADHF | 2.2 | 2.2 | 2.2 |
| Furosemide | 1.7 | 1.7 | 1.7 |
| Spirolactone | 1.7 | 1.7 | 1.7 |
| ACEI/ARB/ARNI | 1.3 | 1.3 | 1.3 |
| Beta-blockers | 1.2 | 1.2 | 1.2 |
| Digitalis | 1.2 | 1.2 | 1.2 |
| Antiplatelet agent | 1.9 | 1.9 | 1.9 |
| Lipid-lowering therapy | 1.9 | 1.9 | 1.9 |
| SGLT-2 | 1.2 | 1.2 | 1.2 |

VIF: variance inflation factor; VIF = 1/(1-R^2^). Abbreviations as in Table ​1.

Note: The variables with VIF>3 will be regarded as collinear variables and cannot be included in the multiple regression model.
